# An adult case of small bowel intussusception caused by hemangioma presenting with intestinal obstruction: A case report

**DOI:** 10.1097/MD.0000000000032268

**Published:** 2022-12-23

**Authors:** Chengyu Shi, Yangyang Yu, Laixiang Zhang, Cheng Gao

**Affiliations:** a Department of Hepatobiliary Surgery, Qingdao, China; b Department of Vascular Surgery, Qingdao Center Medical Group, Qingdao, China.

**Keywords:** adult intussusception, hemangioma, intestinal obstruction, small bowel

## Abstract

Both small bowel intussusception in adults and small bowel hemangioma are rare benign mass lesions. Moreover, a secondary effect of intussusception caused by hemangioma is extremely rare.

**Patient concerns:**

A 87-year-old female suffered from intussusception and intestinal obstruction caused by hemangioma located in the small bowel (February 14, 2020), reporting abdominal distention without nausea and vomiting.

**Diagnosis:**

Emergency abdominal and pelvic computed tomography showed an intussusception with the evidence of associated small bowel obstruction. Histological analysis revealed as small intestinal hemangioma accompanied by mesenteric ulcer.

**Interventions:**

The patient underwent segmental resection of intussusception of intestine instead of invalid conservative treatment.

**Outcomes:**

Although the postoperative pathological results were inconsistent with preoperative imaging examination, the old woman recovered well.

**Conclusion:**

The literature on intussusception of small intestine has described several possible causes including hemangioma, which more likely results in gastrointestinal bleeding or abdominal pain. Yet we experienced a rare case presenting as abdominal distention without nausea and vomiting, Therefore, preoperative diagnosis and localization of these lesions is of great importance. We recommend high resolution contrast-enhanced computed tomography and magnetic resonance imaging should be considered in diagnosis while capsule endoscopy is not available owing to the intestinal obstruction, as long as in facilitating surgical excision.

## 1. Introduction

Although the incidence of small intestinal hemangioma is rare, ranging from 5% to 10%^[1]^ of all small bowel benign lesions, evenly throughout the jejunum and ileum, lack of specific manifestations in early stage. Small bowel hemangioma maybe as 1 feature of Renda–Oslar–Weber Syndrome in accordance with the hemangioma of lung, liver and oral mucosa. We present a case with intussusception surrounded by polypoid mass-hemangioma of small bowel, which was recognized as showing “cuppy sign” on several clinical imagines.

The ethics committee of Qingdao center hospital approved this study, and the written informed consent was obtained from the patient’s legal guardian.

## 2. Case presentation

**Figure 1.**
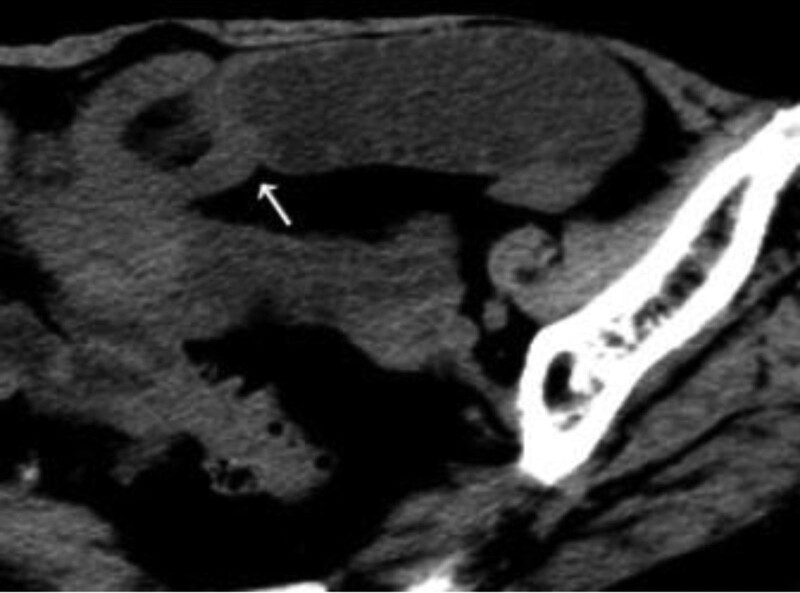
An abdominal computed tomography of small bowl intussusception showed “cuppy sign” (white arrow) presenting with obstruction.

A 87-year-old female had a history of vague pain on upper abdomen for 10 days. She visited our hospital reporting abdominal distention without nausea and vomiting, she had last opened her bowels 1 day before the admission, however, there was no typical history of blood or mucus from the rectum. Emergency abdominal and pelvic computed tomography (CT) showed 1 segment of small intestine was inserted into another, formed the sign of “cuppy” and edema of related intestinal wall located in the part of ileum, suggestive of an intussusception with the evidence of associated small bowel obstruction (Fig. 1). The patient was hospitalized immediately.

On physical examination, the vital signs were stable, the patient had abdominal tenderness beneath the xiphoid process, there were no signs of peritonism. No masses, organomegaly or vascular bruits were detectable.

On admission, analysis of peripheral blood showed mild anemia (red blood cells, 3.48 × 10^9^/L; hemoglobin, 109 g/L; and hematocrit, 0.31 L/L), no abnormality was found from the rest laboratory examination.

The patient was initially managed conservatively with intravenous fluids and nasogastric aspiration, the symptoms failed to improve with these conservative treatment and consequently received a laparoscopic exploration in emergency. Proximal portion of the ileum was discovered in states of distention and engorgement, that was opposite to the distal segment, no obstruction and intussusception were found in the ileocecal junction.

**Figure 2.**
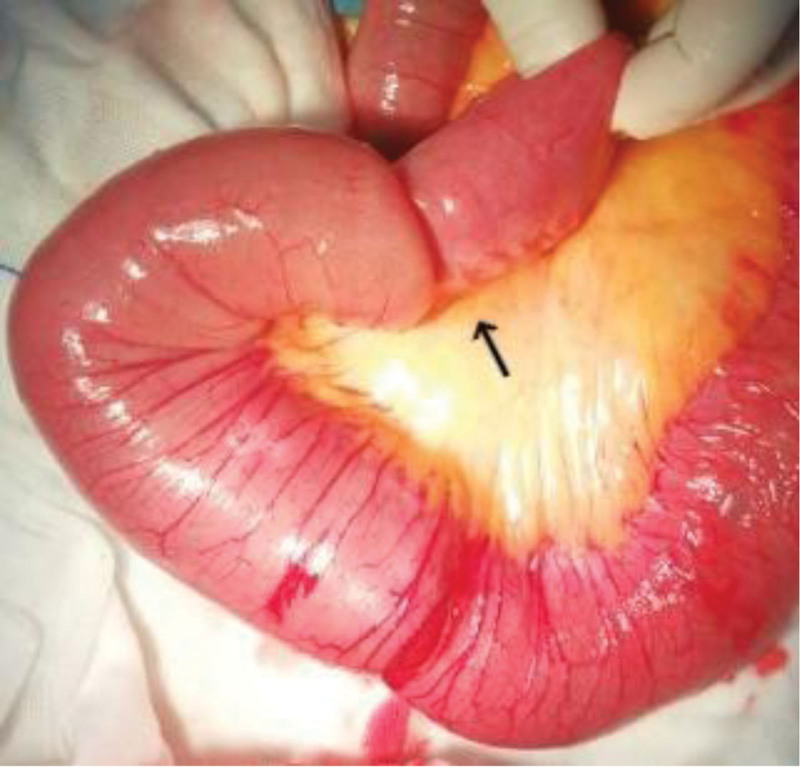
Surgical exploration revealed proximal portion of the bowel and its mesentery into a more distal segment (black arrow).

**Figure 3.**
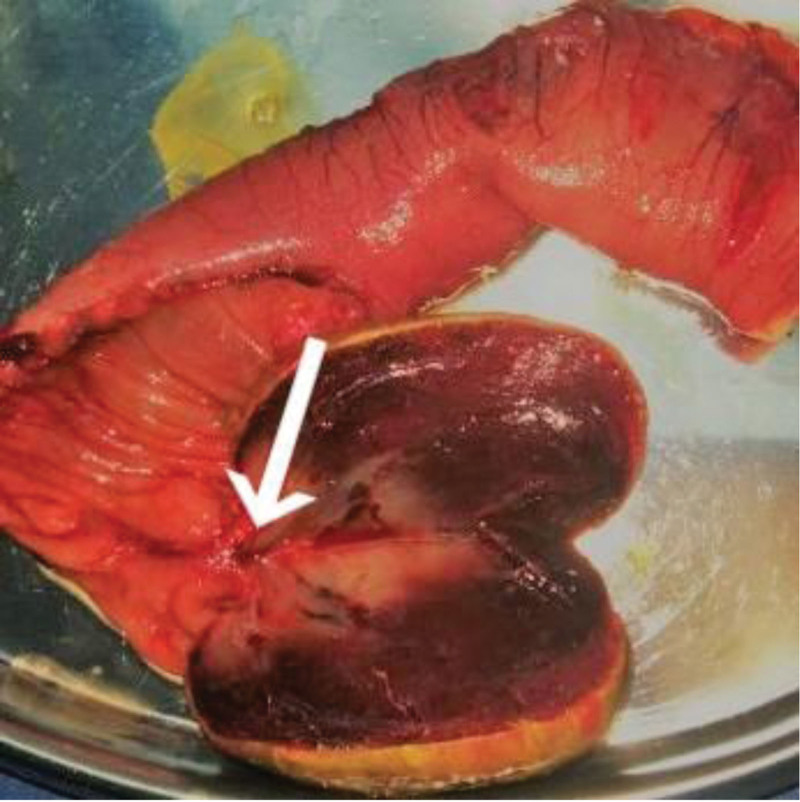
Grossly, the cut surface of tumor showed a gray brown polypoid, with a pedicle connected to the mesentery of ileum (white arrow).

**Figure 4.**
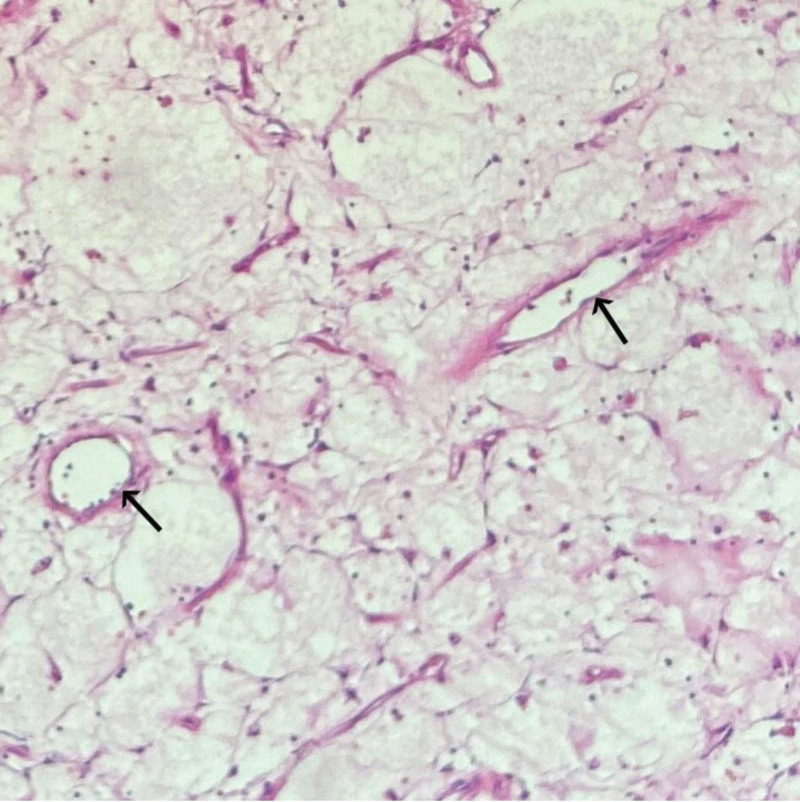
The proliferative vessels are mostly composed of small-sized thin-walled vessels lined by bland looking plump and ovoid (or irregular) endothelial cells (black arrow).

Segmental resection of intussusception of intestine was performed (Fig. 2). Resected gross specimen showed a section of small intestine, about 16-cm long, there was a gray brown polypoid tumor with the size of 5 × 4 × 3 cm, 16-mm away from the cutting edge of one side, 14-cm from the edge of the other side. The tumor had a pedicle of 5-mm in length. The section of the tumor was grayish brown and tough. No abnormality was found on the other mesenteric and serous surfaces. Histological analysis revealed as small intestinal hemangioma accompanied by mesenteric ulcer. Chronic inflammation and hyperemia were found in bilateral incisional margin mucosa (Fig. 3). Histological analysis was in Figure 4 as followed.

Surgical intervention adherence exactly met the need of the patient. Postoperative pain scores (NRS 2002) ranged from 0 to 4, which proved good tolerability for the patient. No adverse and other unanticipated events occurred in hospital.

## 3. Discussion

The definition of intussusception is mentioned above (Figs. 1 and 2): invagination of a proximal portion of the bowel and its mesentery (intussusceptum) into a more distal segment (intussuscipiens),^[2]^ meanwhile, it can result in impaired peristalsis, obstruction, and even vascular compromise directly.^[3]^ Intussusception of small intestine is a rare disease in adult patients while primarily in children. Childhood intussusception is idiopathic in 95% of cases while adult intussusception is secondary to intraluminal lesion, has a specific pathological changes – in general, all kinds of malignant or benign neoplasm.^[4]^ For example, intussusception of the large intestine occur more frequently secondary to malignant lesions: adenocarcinoma and lymphoma.^[5]^ However, a secondary effect of intussusception caused by small bowel hemangioma is extremely rare. Few cases reported of small bowel intussusception were hemangioma in origin.

Intestinal hemangioma is not a true tumor but one of the congenital benign vascular malformation lesions, which are rarely found in adult patients. These benign malformations are classified into 4 categories^[6]^: Capillary lymphangioma, cavernous lymphangioma, cystic lymphangioma (hygroma), and hemolymphangioma (a combination of hemangioma and lymphangioma). All kinds of the categories may result in occult or massive gastrointestinal (GI) bleeding, perforation, abdominal pain, obstruction and intussusception,^[7]^ According to the location of the overlapping organs, intestinal overlap presents a variety of nonspecific clinical symptoms, GI bleeding and abdominal pain are the most common,^[8]^ leading to anemia in most patients.^[9]^ The unspecific symptoms of the early clinical manifestations in small intestinal hemangioma due to the particularity and complexity of the small intestinal anatomy. Small intestinal hemangioma is usually occult in onset, most of the patients are in a progressive stage once they have the typical symptoms,^[10]^ as a result, it is difficult to characterize with laboratory testing, meanwhile, it is hard to detect lesions with multiple inspections because of the concealed location of jejunum.

Although early diagnosis for small bowel hemangioma is quite difficult. However, several imaging modalities including wireless capsule endoscopy, double balloon push-type enteroscopy or colonoscopy, multiphase computed tomography enterography, magnetic resonance enterography, double barium contrast gastrointestinal imaging are currently available options to investigate small bowel lesions.^[11]^ Small bowel capsule endoscopy is a noninvasive imaging test and can be recommended when the source of bleeding remains unidentified after upper and lower endoscopy.^[12–14]^ The entire small intestine lesions including intraluminal ulcer, submucous polypus, parasite and foreign matter can be observed clearly, while capsule endoscopy also has limitations, such as biopsy, inaccurate positioning, not suitable for large amount of bleeding or intestinal obstruction. When positive findings are obtained on capsule endoscopy and there is no evidence of obstruction, double balloon enteroscopy or push enteroscopy is recommended for further management.^[14]^ As are also recommended in the American College of Gastroenterlogy clinical guideline (edition 2015).^[15]^

Despite the capsule endoscopy, contrast-enhanced CT and magnetic resonance imaging (MRI) play very important roles in the diagnosis of the lesions.^[16]^ The MRI sign of “filling-defect” image developed from small bowel neoplasm through oral iodine contrast, a relatively low-density shadow in T1 image while high-density shadow in T2 image. In this case, although a typical intussusception CT sign of “cuppy” and intestinal obstruction suggested the space occupying lesion in small bowel, however, the indefinite diagnosis was made due to the absence of 3-dimensional reconstruction of the small intestine. Therefore, we suggested that radionuclide scanning and selective angiography should be performed in order to confirm the diagnosis and further therapeutic schedule when endoscopy is not available (because of the intestinal obstruction). CT virtual endoscopy has been used to diagnose small intestinal hemangioma in domestic and abroad, but it has not been widely carried out in large medical centers.^[17]^

Once the disease is diagnosed, it is appropriate for patients to receive surgical therapy. Laparoscopic or laparotomy exploration should be enforced based on the pathogenetic condition in case the indefinite diagnosis was made, thorough excision of the lesions is the ideal treatment. For a single lesion or dense lesions confined to 1 segment of intestine, the therapeutic effect can be achieved by intestinal resection. It is necessary to prevent postoperative short bowel syndrome and nutritional absorption disorder when large-scale resection of multiple lesions is performed.

In conclusion, we experienced a rare case with small bowel intussusception caused by hemangioma, which is a benign vascular malformation that can cause chronic GI bleeding and intestinal obstruction presenting as chronic abdominal pain and distention. Therefore, preoperative diagnosis and localization of these lesions is of great importance. We recommend high resolution contrast-enhanced CT and MRI should be considered in diagnosis while capsule endoscopy is not available owing to the intestinal obstruction, as long as in facilitating surgical excision. In addition, capsule endoscopy and further double balloon enteroscopy or push enteroscopy for the diagnosis and management of obscure GI bleeding are considered as the 1st-line procedure, also the 1st-line initial examination of small intestine.

## Author contributions

**Data curation:** Yangyang Yu, Laixiang Zhang.

**Formal analysis:** Laixiang Zhang.

**Resources:** Chengyu Shi.

**Supervision:** Cheng Gao.

**Writing – original draft:** Chengyu Shi.

**Writing – review & editing:** Chengyu Shi.
